# Relationship among some coordinative and dynamic strength capabilities and constructive and conceptual thinking among preschool-age children

**DOI:** 10.3389/fpsyg.2024.1349884

**Published:** 2024-03-14

**Authors:** Chipo Malambo, Adéla Klepačová, Kateřina Brodská, Cain Craig Truman Clark, Martin Musálek

**Affiliations:** ^1^Faculty of Physical Education and Sport, Charles University, Prague, Czechia; ^2^College of Life Sciences, Birmingham City University, Birmingham, United Kingdom

**Keywords:** motor coordination, constructive thinking, conceptual thinking, cognitive development, preschool children

## Abstract

**Background:**

Existing research underscores the positive influence of consistent physical activity, fitness, and motor coordination on school-aged children’s cognitive and academic performance. However, a gap exists in fully understanding this relationship among preschoolers, a critical age group where the development of cognitive functions is significant. The study aims to expand upon existing evidence that connects motor and cognitive development by examining the correlation between specific motor coordination and physical fitness skills and the development of constructive and conceptual thinking in preschool-aged children.

**Methods:**

Data from 56 children aged 4–5 years (mean age 4.5 ± 0.36y), comprising 30 girls and 26 boys, participated in this study. We assessed muscular strength (via standing long jump, wall toss test, flexibility), agility (4 × 5 m shuttle), cardiorespiratory fitness (20 m pacer test), and motor coordination (lateral jumping, platform shifting). Cognitive abilities were measured using the IDS-P.

**Results:**

Linear regression models showed that significant predictors of constructive thinking scores were observed solely for flexibility (*p* = 0.02) and shifting platforms (*p* = 0.01). Notably, flexibility exhibited a negative relationship (*β* = −1.68). In the context of conceptual thinking, significant predictors (*p* < 0.05) included standing long jump (*p* = 0.01), jumping laterally (*p* = 0.005), shifting platforms (*p* = 0.001), throwing (*p* = 0.02).

**Conclusion:**

Coordination-demanding activities seem to be related considerably to conceptual thinking in preschoolers. Integrating such motor activities into preschool curricula that demand cognitive engagement can positively influence the development of cognitive functions.

## 1 Introduction

Research repeatedly shows the ability for improvement of cognitive and academic performance in school-aged children through regular involvement in aerobic exercise and perceptional motor training, exemplifying the benefits of Fundamental movement skills (FMS), physical activity (P.A.), physical fitness (P.F.), and Motor coordination (MC) (Wick et al., 2021; Albuquerque et al., 2022). However, researchers have documented steadily declining FMS, PA, P.F., and MC (Tomkinson et al., 2019). This decline has been attributed to several factors such as social, behavioral, physical and physiological tenets (Tomkinson and Olds, 2007), including budget cuts in physical education programs (Pühse and Gerber, 2005), despite the suggested evidence pointing to the possible significant relationship between motor and cognitive development possibly from preschool-aged children (Wick et al., 2021).

The preschool period between 3 and 5 years of age is developmentally essential. Indeed, preschoolers show rapid growth and changes in areas of cognitive development, physical development, and neurodevelopment (Gerber et al., 2010). Noninvasive structural and functional neuroimaging methods have shown that Early childhood witnesses profound structural and functional changes in the brain, including cortical area expansion, cortical thickness reduction, and varied subcortical volume alterations. Cognitive tasks involve more brain regions in younger individuals, suggesting early “scaffolding” mechanisms that fade with age. The preschool years signify dynamic growth and cognitive development, facilitated notably through experiences (Brown and Jernigan, 2012). These early experiences lead to the pruning of dendrites in neurons. Furthermore, myelination plays a significant role in the development of children’s abilities and capacities that we observe.

The development of fatty myelin around neurons’ axons in children results in enhanced cognitive processing characterized by increased speed, coordination, and complexity. Myelination facilitates efficient communication among neurons, enabling the execution of coordinated behaviors (Mabbott et al., 2006; Dubois et al., 2014). The timing and distribution of myelination patterns align closely with initiating and refining cognitive functions and behaviors. Initially, myelination primarily occurs in brain regions responsible for sensory and motor functions. During early childhood, children demonstrate rapid information processing abilities, enabling them to perform intricate sequences of physical actions, such as catching and throwing a ball. Moreover, they display improved cognitive skills, adequately retaining and responding to inquiries over extended durations. Furthermore, the role of experience in this process is notable. Children develop familiarity through repeated engagement in activities, allowing for quicker task execution and multitasking capabilities (Merzenich, 2001; Mabbott et al., 2006; Brown and Jernigan, 2012; Dean et al., 2014; Dubois et al., 2014).

Additionally, play as part of activities is a mechanism through which experience is achieved for this age group. Piaget previously asserted the importance of how a child’s psychological development occurs through action, which leads to the development of thinking and perception (Piaget, 1952). In his theory of cognitive development, Piaget mentions the importance of motor activity in developing aspects of intelligence, such as operational Intelligence (Lawrence, 1957).

Physical activity has been shown to contribute to better outcomes in children’s learning and is regarded as essential for operational Intelligence (Piaget, 1952; Norris et al., 2020). The arguments raised are that movement, as seen through play/P.A. in children, has several positive impacts on cognition through several mechanisms, such as glucose delivery, angiogenesis, and neurotransmitter levels, which can stimulate cognitive development in preschool children (Álvarez-Bueno et al., 2017; Norris et al., 2020). Some researchers have also suggested that the increase in oxygenation is due to the rise in blood flow (Iughetti et al., 2011). Furthermore, there is an increase in brain-derived neurotrophic factor (BDNF), which is vital for maintaining and promoting neural development. This increase in BDNF is important in children and adolescents as it supports brain development and plasticity (Gómez-Pinilla et al., 1998; Iughetti et al., 2011). For instance, Veraksa et al. (2021) highlighted the importance of physical fitness in their study, showing that Inhibitory control and working memory were positively linked with tests of physical fitness (coordination, speed and coordination demanding activities).

Motor coordination refers to the degree of proficiency in performing various movements, such as gross (e.g., jumping) and fine (e.g., manual dexterity or precision) motor skills, necessitated by the nervous system and muscles, and also reflects physical fitness (Haga and Haga, 2008; Walhain et al., 2016; Utesch et al., 2019). Physical fitness is commonly understood as the ability to carry out daily tasks with vigor and alertness, without undue fatigue and with ample energy to enjoy leisure-time pursuits and to meet unforeseen emergencies (Caspersen et al., 1985). However, physical fitness in this paper is to be understood in two broad groups: health-related fitness (aerobic fitness, muscular strength, muscular endurance, and flexibility) and skill-related fitness (agility, balance, coordination, power, reaction time, and speed) (Molnár and Livingstone, 2000).

In one systematic review (Donnelly et al., 2016), physical activities (i.e., P.E. play, sport, exercise.), fitness, cognitive function, and academic achievement among 5 to 13-year-olds found that most research supports the positive influence of physical activity on children’s cognitive functioning and brain processes. A more recent review on the same topic and similar age groups found that increased time allocated to physical activities was positively associated with academic performance (James et al., 2023). Additionally, it has been suggested that the type of activity may not be the primary factor; instead, the physiological changes induced by physical activity play a crucial role in improving preschool-aged children; although limited, some data is available on academic performance (Bass et al., 2013).

In preschool-aged children, although limited, there is some data available. In 2002, Planinsec found that the motor dimensions with the strongest associations with cognitive abilities were coordination and the speed of movement (Planinsec, 2002). Another study among preschool children found that coordination is related to attention in preschool children. Thus, competent performance in complex motor skills (i.e., hopping on one leg) is posited to predict attention in preschool children (Wick et al., 2022). Similarly, in 2005, Voelcker-Rehage reported significant moderate associations between reaction time (*r* = 0.41), coordination (*r* = 0.30), and fine motor skills (*r* = 0.34) and cognitive performance (i.e., visual processing) in 4- to 6-year-old children (Voelcker-Rehage, 2005). Additionally, another study showed that different facets of physical fitness were associated with aspects of cognitive function. For instance, higher baseline aerobic fitness and motor skills were related to better spatial working memory or attention at baseline and, to some extent, to their future improvements over the following 9 months (Niederer et al., 2011). While not exclusively focused on preschool-aged children, a systematic review by van der Fels et al. (2015), which included children of that age, explored the relationship between motor skills and cognitive abilities in typically developing children aged 4–16. The findings revealed moderate to strong correlations between fine motor skills, bilateral body coordination, and speed of movement (e.g., foot tapping, running in a zigzag) and cognitive performance, encompassing memory, visual processing, and executive functions.

While these studies show a positive association of physical fitness and motor skills with cognitive performance, shuttle running and throwing with inhibition (Gavrilova et al., 2022); inhibitory control, both visual and verbal working memory with shuttle running and throwing (Veraksa et al., 2021); fine coordination, reaction speed, action speed, jump power, fine coordination, balance and cognitive function (Voelcker-Rehage, 2005; Veraksa et al., 2021; Gavrilova et al., 2022), the role of the motor coordination, physical fitness and their components in cognitive development remain an area that could benefit with more evidence. Although Chang et al. (2013) found that motor skills can predict some aspects of cognitive skills in preschool, Houwen et al. (2017) found weak associations between the two concepts. A few existing studies (Mavilidi et al., 2015; Cook et al., 2019) have examined motor domains like FMS, P.F., and P.A. concerning cognitive function in preschool-aged children. However, these studies have primarily concentrated on the sum scores of these concepts, making it challenging to assess actual performance on their components. In our systematic review, we underscored these limitations alongside methodological issues such as reliance on self-reported data and subjectively assessed data Malambo et al. (2022).

In this study, we have limited our scope of cognitive functions to two: constructive and conceptual thinking. Constructive thinking refers to a set of cognitive, productive and counterproductive automatic habitual thoughts that affect one’s ability to think in a manner that solves problems (Epstein, 1992). Individuals who are good at constructive thinking are better at managing their emotions. Constructive thinking is also a powerful predictor of problem-solving and success (Sternberg and Wagner, 1986; Epstein, 1992). Good constructive thinkers possess a set of ingrained adaptive beliefs that support their ability to regulate their emotions and maintain an action-focused coping style. Poor constructive thinkers, on the other hand, have maladaptive automatic ideas that obstruct the process of emotion monitoring.

Meanwhile, conceptual thinking categorizes things in the world, mainly representing groups with something in common (Oakes and Kovack-Lesh, 2013). Concepts allow us to infer unknown entities’ potential affiliation to existing categories. The ability to categorize and conceptualize is essential to human cognition because, without them, it would be impossible to comprehend perception, memory, language, or any other form of general cognition (Harnad, 2017).

As previously mentioned, children learn through play. As far back as the 60s, Piaget (1962) to children’s intelligence development. His theory of play argues that as the child matures, their environment and play promote further cognitive and language development. Children’s play involves pretend play and physical activity play (Pellegrini and Smith, 1998). Through this, children’s conceptual and constructive thinking processes develop, among other cognitive processes. Research has shown that when young children are taught basic relational concepts, they show improvement in understanding these concepts on standardized achievement tests (Piersel and McAndrews, 1982; Zhou et al., 2000; Boehm, 2013). However, basic, relational concepts are complex for many children to grasp because they have no constant referent. On the other hand, some suggest that engaging in movement activities can facilitate the development of these cognitive skills (Wick et al., 2022).

As previously noted, there is evidence that motor skills are associated with cognitive development and that interventions that improve motor skills can also improve cognitive development. However, insufficient evidence shows to what extent tests of motor coordination and aspects of P.F. can facilitate constructive and conceptual thinking in preschoolers, considering it might be necessary during their development. Therefore, building upon the premise that intellectual engagement increases with more significant coordination challenges and motor task complexities, as Horga (1993) highlighted, our study aimed to explore the relationships among some capabilities of motor coordination, physical fitness, and constructive and conceptual thinking. Building upon existing literature, we hypothesized that motor coordination and physical fitness tests, demanding precise coordination and engaging high-order cognitive functions, strongly relate to conceptual and constructive thinking. This hypothesis is grounded in the observation that motor activities like lateral skipping and shifting platforms demand a certain level of motor proficiency, encompassing decision-making processes, planning, intellectual components, and rhythmicity - timing. We believe integrating these elements constitutes a cohesive strategy for executing coordination-demanding movements, a viewpoint congruent with Bernstein’s explanation (Bernstein, 1996).

## 2 Methods

### 2.1 Participants

An exploratory study design examined preschoolers’ physical fitness and cognitive performance from a convenience sample of five kindergartens in Prague, Czech Republic. Fifty-six children aged 4–5 (mean age 4.5 ± 0.36), comprising 30 girls and 26 boys, participated in this study. The Faculty of Physical Education and Sports Ethics Committee approved the study and consent procedures. Before the start of the study, parents or legal representatives received written information on the study’s aims and design, including potential risks and benefits. Parents or legal representatives of all participating children provided their written informed consent before the study started. The study was conducted between March 1 and June 30, 2021.

### 2.2 Procedure

Before the testing phase, arrangements were made to establish testing conditions, with children participating in small groups, typically about five children per task. All participants engaged in a standardized 10-min general warm-up session before commencing the tests. Examiners received prior training to ensure consistent administration, adhering to the following standardized procedures: proficient demonstration of each test technique with verbal explanations (a), allowing participants to practice each task before test administration (b), emphasizing that children should perform each task to their best abilities (c), and providing motivational feedback while refraining from verbal feedback on skill performance (d).

The assessment commenced with the collection of anthropometric measurements, encompassing height and weight. Height was measured using a mobile anthropometer A-216, with precision to the nearest 0.1 cm, while weight was measured using a scale to the nearest 0.1 kilogram. Cognitive assessments were prioritized to mitigate fatigue, followed by motor coordination and physical fitness evaluations. The evaluations culminated in activities such as the 20-meter endurance run.

### 2.3 Test measurements

#### 2.3.1 Motor screening

In this study, we conducted measurements encompassing motor coordination and physical fitness. We aimed to quantify elements comprising both a muscular component (including power or explosive strength, isometric strength, and muscular endurance) and a motor skill component (such as agility, balance, coordination, and speed of movement), as outlined in prior studies (Bouchard and Shephard, 1994; Malina et al., 2004). This study addresses both dimensions; the children participated in seven tests encompassing physical fitness and motor coordination capabilities.

Considering the nature of our study and the target sample, we meticulously chose age-appropriate test items tailored to this population. We aimed to assess specific tests rather than composite scores, focusing on measurements that gauge particular capabilities rather than employing entire test batteries. For assessing motor coordination, we included items such as *Jumping laterally* and *Shifting platforms* as seen in the Motor competence assessment (MCA) (Luz et al., 2016) and Körperkoordination Test für Kinder (KTK) (Kiphard and Schilling, 1974). For physical fitness and endurance, we chose the *20 m endurance run, Standing long jump* - (Cadenas-Sanchez et al., 2016), the *Wall toss test* -MCA (Luz et al., 2016), *Shuttle run 4x5m* (Milosevic and Petrovic, 2015) and *sit and reach* measuring flexibility (Musálek et al., 2021).

#### 2.3.2 Motor coordination tests

1. *Jumping laterally* – the child makes consecutive jumps from side to side over a small beam (60 cm × 4 cm × 2 cm) as fast as possible for 15 s. The child is instructed to keep their feet together; the number of correct jumps is recorded.
2. *Shifting platforms* – involves the child starting with both feet positioned on one platform (measuring 25 cm × 25 cm × 2 cm, supported on four legs 3.7 cm high) while holding a second identical platform in their hands. They are instructed to place the second platform beside the first one and step onto it. Subsequently, the first platform is lifted and positioned beside the second, allowing the child to step onto it. This sequence continues for a duration of 20 s. Each successful transfer from one platform to the other earns two points: one for shifting the platform and another for transferring the body. The total number of points accumulated within the 20-s timeframe is recorded. If the child falls off during the process, they are instructed to get back onto the platform and continue the test.

#### 2.3.3 Physical fitness and endurance tests

1. *20 m endurance run* - Cardiorespiratory fitness was evaluated using a modified version of the original 20 m shuttle run test, termed the PREFIT 20 m SRT. In this assessment, participants were required to shuttle back and forth between two lines set 20 meters apart, prompted by an audio signal. The test concluded if the child failed to reach the end lines synchronously with the audio signal on two consecutive occasions or if the child ceased due to exhaustion. We fully adopted the PREFIT version, wherein the original PREFIT version 20M pacer test commences at a speed/velocity of 6.5 km/hour. Due to the children’s young age, who may not be familiar with the concept of tempo, we utilized a pacemaker to assist in maintaining the required pace. These adaptations included reducing the initial speed and having two evaluators accompany a smaller group of children (ranging from 4 to 8 preschoolers of the same age) to ensure an appropriate pace (Cadenas-Sanchez et al., 2016).
2. S*tanding Long Jump* (SLJ) – placed below a starting line, the participant must jump as far as possible, using both feet simultaneously on the take-off and landing. The jump is made over a surface with marked measuring lines or a measuring tape placed on one side of the free jumping space, perpendicular to the starting line. The distance (in cm) is measured between the starting line and the place where the back of the heel is closest to the starting line. The final score is the best of 3 correct trials.
3. *Wall toss test* -The wall toss test involves standing below a 1 m line marked on the floor, positioned at least 6 m away from a wall measuring at least 5 m × 5 m. Participants execute an overarm action to throw a ball with maximum force against the wall. A cross measuring 40 cm × 40 cm, situated midway on the wall and 170 cm from the ground, serves as the target. Children aged 3–10 years use a tennis ball (diameter: 6.5 cm; weight: 57 g), while those aged 11 and older employ a baseball ball (diameter: 7.3 cm; weight: 142 g). The ball’s velocity at its peak is measured in meters per second (m/s) using a velocity radar gun positioned beside the participant’s dominant hand, near the floor line, at approximately shoulder level, and facing the target wall (outbound). The final score is determined by selecting the best performance out of three correct trials.
4. *Shuttle run 4x5m* - Children started to run from a starting line from standing position on signal Ready-steady-GO! Each child had to run four times the distance of 5 meters, which was determined by two color cones, as fast as possible. A trained instructor ran with the children to motivate them to achieve their maximum speed potential. At the end of each track, the child had to touch the top of the color cone and run back as quickly as possible. Each child had a trial run, after which each participant had two attempts with a five-minute rest between each attempt. The time the participant needed to run the whole four tracks was recorded. The fastest time on 0.1 s was recorded.
5. *Seat and reach test* - The Sit and Reach test for preschool children utilized a modified bench with a height of 25 cm, differing from the original 30 cm version (source: https://lafayetteevaluation.com/products/121086-sit-reach-box, accessed on September 22 2021). During the test, the child was instructed to sit against a wall, straighten their legs and back, and lean against the wall with their entire back. A bench was positioned against the child’s legs so that their feet rested on one side of the bench. The child then extended their arms forward, and the metric scale on the bench was aligned with the tip of their middle finger. Following this, the child performed a forward bend, ensuring not to bend the knees. The examiner monitored the child’s knee position throughout the test. The maximum distance the child reached without breaking the rules was recorded. The entire test was performed twice in quick succession.

#### 2.3.4 Cognitive test

Constructive thinking and conceptual thinking were evaluated using the IDS-P (Hagmann-von Arx et al., 2018). This test battery is a revised version of the Intelligence and Development Scale (Grob et al., 2009). The IDS showed high reliability and validity (Hagmann-von Arx et al., 2008, 2012, 2013), whereas the IDS-P has strong construct validity (Grieder and Grob, 2020). The IDS-P assesses cognitive ability and growth in several fundamental areas (e.g., executive functions, psychomotor skills, social–emotional competencies, scholastic skills and attitudes toward work).

1. *Constructive Thinking*: The child uses triangular and rectangular tiles to assemble various geometric configurations. Each configuration is presented to the child individually, accompanied by a set of tiles precisely corresponding to the requirements for constructing the specified shape. This evaluative framework encompasses a total of 12 distinct geometric configurations. The test administration is concluded if the child fails to respond correctly to three consecutive tasks. Importantly, no time constraint is imposed upon the child during this evaluation.
2. *Conceptual Thinking*: The child is presented with three distinct original images characterized by a shared attribute. Subsequently, the child is given five additional images and tasked with identifying and selecting two images that exhibit the same common feature as the original set. This cognitive assessment comprises a total of 11 discrete tasks. The test administration is concluded if the child fails to respond correctly to three consecutive tasks. Importantly, no time constraint is imposed upon the child during this evaluation.

### 2.4 Statistical analysis

All analyses were performed using R (R Core Team, 2021). Firstly, for descriptive analyses, measures of age, sex, height, weight, and MC are presented as means (standard deviations, S.D.). Initially, descriptive analyses were performed, showing age, sex, height, weight, and motor coordination (MC) P.F. tests and means with corresponding standard deviations (S.D.). Later, multiple linear regression (MLR) was used. In this work, the MLR model was implemented to predict the value of dependent variable Y (constructive and conceptual thinking) starting from the knowledge of several independent variables [Age, sex, body mass index (BMI), height, standing long jump, jumping laterally, shifting platforms, wall toss test (right and left, 20 m endurance run, shuttle run 4 × 5 m and sit and reach tests]. The level of significant α is equal to 0.05.

## 3 Results

The study sample comprised 56 children, 30 girls and 26 boys, with an average age of 4.5 years (SD = 0.36). Anthropometric measurements revealed that the children had an average weight of 17.6 kilograms (SD = 2.87) and an average height of 107.2 centimetres (SD = 5.29). There were no significant mean differences in sex in performance in MC and P.F. tests and cognitive variables in our initial analysis. The sample characteristics are summarised in Table 1.

**Table 1:** Descriptive of children’s characteristics and scores on coordinative, physical fitness and cognitive assessments.

**Age and anthropometry**	**Both sexes**	**Boys**	**Girls**
Age [years], mean (SD)	4.5 (0.36)	4.6 (0.29)	4.5 (0.41)
Weight {Kg}, mean (SD)	17.6 (2.87)	18.3 (3.53)	17.0 (2.02)
Height [cm], mean (SD)	107.2 (5.29)	108 (5.46)	106 (4.96)
**Motor coordination physical fitness tests**			
Standing long jump [cm], mean [SD]	86.3 (15.81)	90.2 (16.74)	82.9 (14.38)
Shuttle run 4 × 5 meters [s], mean [SD]	10.8 (3.10)	10.8 (1.73)	10.9 (1.35)
Sit and reach test [cm], mean [S.D.]	18.05 (5.62)	16.1 (6.06)	19.7 (4.69)
Jumping laterally [n], mean [S.D.]	9.7 (2.78)	11.1 (3.95)	10.8 (3.41)
Shifting platforms [n], mean [SD]	9.7 (2.78)	9.4 (2.83)	9.97 (2.74)
Wall toss test (Right Hand) [Speed], mean [S.D.]	6.8 (1.72)	7.8 (1.65)	5.9 (1.25)
Wall toss test (Left Hand) Speed, mean [SD]	5.9 (1.50)	6.8 (1.14)	5.3 (1.43)
20 endurance run [Speed], mean [SD]	16.1 (7.7)	15.2 (6.52)	16.9 (8.69)
**Cognition**			
Constructive thinking [no. of points], mean (S.D.)	10.6 (2.34)	10.9 (2.21)	10.3 (2.45)
Conceptual thinking [no. of points], mean (S.D.)	9.2 (2.80)	9.5 (3.11)	8.8 (2.52)

Table 2 shows the results of multiple linear regression models to identify the key predictors of constructive and conceptual thinking scores in the study’s cohort of children. For constructive thinking, the model incorporated age, sex, BMI, height, and various physical fitness tests as predictors. Only flexibility and shifting platforms showed significant associations (*p* < 0.05) with constructive thinking scores. Flexibility exhibited a negative relationship (β = −1.68), while shifting platforms demonstrated a positive association (β = 3.73). In the case of conceptual thinking, the model considered the same set of predictors. Standing long jump, jumping laterally, shifting platforms, throwing right, and throwing left were statistically significant (*p* < 0.05) predictors, with shifting platforms showing the strongest positive relationship (β = 6.17).

**Table 2:** Multiple linear regression analysis of coordinative, physical fitness tests to constructive and conceptual thinking.

**Dependent variable**	**Predictors**	***β***	***t***	***p***	***R*** ^***2***^	**Adjusted *R*** ^**2**^
**Constructive thinking score**	Age	−1.52	−1.07	0.22	0.29	0.08
	Sex	−9.03	1.07	0.29		
	BMI	3.22	1.12	0.23		
	Height	−8.18	−1.03	0.31		
	Standing long jump	−1.00	−0.36	0.71		
	Shuttle run 4 × 5 m	−4.11	−0.02	0.99		
	Sit and reach	−1.68	−2.49	0.02*		
	Jumping laterally	6.76	0.06	0.95		
	Shifting platforms	3.73	2.53	0.01*		
	Wall toss test (Right Hand)	−3.48	−1.24	0.22		
	Wall toss test (Left Hand)	2.11	0.68	0.50		
	20 m endurance run	−4.34	−0.97	0.33		
**Conceptual thinking score**	Age	−9.95	−0.74	0.94	0.39	0.22
	Sex	−6.87	−0.74	0.47		
	BMI	−1.46	−0.49	0.62		
	Height	1.32	0.15	0.88		
	Standing long jump	7.79	2.58	0.01*		
	Shuttle run 4 × 5 m	3.63	1.11	0.27		
	Sit and reach	6.99	0.94	0.35		
	Jumping laterally	−3.74	−2.94	0.005***		
	Shifting platforms	6.17	3.79	<0.000***		
	Wall toss test (Right Hand)	7.13	2.30	0.02*		
	Wall toss test (Left Hand)	−8.52	−2.51	0.02*		
	20 m endurance run	−7.70	−1.55	0.13		

* < denotes significance at <0.05, ** < denotes significance at <0.01, *** denotes significance at <0.001.

## 4 Discussion

Some studies among preschool-aged children have shown that motor domains such as physical activity, physical fitness and fundamental movement skills can predict cognitive function among this age group (Mavilidi et al., 2015; Veraksa et al., 2021; Wick et al., 2022). The present study examined some capabilities of motor coordination and physical fitness and their relationship to constructive and conceptual thinking. We found that shifting platform (motor coordination) and the reach and sit test (measuring flexibility) were significantly associated with performance in constructive thinking. On the other hand, conceptual thinking had more significant predictors (*p* < 0.05): shifting platform, jumping laterally (motor coordination), standing long jump, and wall toss test (both right and left) measuring physical fitness (dynamic strength for lower and upper body).

While some studies have explored the link between physical activity, fitness, and cognitive performance in children, none have investigated the specific relationships between individual aspects of motor coordination and physical fitness to both constructive and conceptual thinking in preschoolers. This unique focus limits our ability to compare our findings with existing research directly. However, studies examining composite scores of various physical capabilities (P.A., P.F., FMS, MC) and broader cognitive domains (inhibition, memory, attention, flexibility) do show significant correlations(Mavilidi et al., 2015; Cook et al., 2019; Albuquerque et al., 2022).

Firstly, per the study hypothesis, our results show motor coordination is related to constructive thinking. We assessed shifting platforms, a complex and demanding exercise, measuring coordination, agility, reaction time and balance control. Such a task requires one to utilize high cognitive functions. The capability to perform a physical or cognitive task swiftly and smoothly hinges on the capacity to suppress irrelevant stimuli originating from the environment (Invernizzi et al., 2017). This ability to block distractions, known as inhibitory control, also influences decision-making. It bridges the gap between cognitive processes and emotions, forming a crucial aspect of constructive thinking (Epstein, 1992). These mechanisms lead to co-activations of different parts of the central nervous system, which may stimulate motor performance and could explain the associations in our study. Although not measuring constructive thinking, other studies similarly show that activities involving precise coordination require a high level of executive thinking, such as working memory and inhibitory control (Voelcker-Rehage, 2005; Gavrilova et al., 2022).

Interestingly, our study found a negative association between a flexibility measure (sit-and-reach test) and constructive thinking. However, we agree that flexibility is behaviorally different from other aspects of motor performance or physical fitness (Fleishman, 1964; Corbin, 1984). Moreover, in this age group, flexibility performance might be more closely linked to growth and changes in the ratio between trunk and limb length, which is typical for preschool age (Bogin, 1990). In addition, Several studies investigating flexibility, cognitive aspects, and academic achievement in children and adolescents (Eveland-Sayers et al., 2009; Kalantari and Esmaeilzadeh, 2016; Moradi et al., 2019) did not observe significant relationships.

Secondly, our results also showed that some aspects of motor coordination (shifting platforms, jumping laterally) and physical fitness and endurance (standing long jump, wall toss right and left) were significantly related to conceptual thinking. As previously stated, it is unsurprising that coordination demanding activities are related considerably to conceptual thinking because of how cognitively demanding they can be, as supported by previous studies (Wick et al., 2022). Concerning physical fitness, both Veraksa et al. (2021) and Gavrilova et al. (2022), although not measuring conceptual thinking, show that throwing both right and left (wall toss test) and standing long jump is associated with cognitive function, which agrees with our findings. Studies demonstrate strong connections between specific coordinate activities and certain cognitive functions (Wick et al., 2022). As hypothesized, more complex and demanding exercises requiring precise coordination showed stronger links to cognitive skills than straightforward tasks. This aligns with previous research highlighting significant connections between specific elements like bilateral coordination, movement speed, and agility with cognitive skills like fluid intelligence and attention in this age group (Planinsec, 2002; Niederer et al., 2011).

Understanding our findings hinges on the concept of “concepts.” Many assessed activities demanded existing knowledge in children. More practice with these activities could translate to a better understanding of those concepts. Conceptual thinking allows us to categorize and form ideas, connecting new things to what we already know. This ability is crucial for human cognition. Without it, grasping various cognitive processes like perception, memory, language, and general cognition would be significantly hindered (Oakes and Kovack-Lesh, 2013; Harnad, 2017). It is important to note that the children in our study received instruction and practice on the activities. Recognizing that understanding requires attention and repetition, this highlights the role of instruction and practice in shaping conceptual development. This underscores the need for further research on the complex interplay between motor skills such as motor coordination, P.F. and endurance, other cognitive abilities like attention and memory, and their combined influence on conceptual thinking.

Increasing the time dedicated to physical activities or physical education in schools and at home could potentially enhance children’s constructive and conceptual thinking. This exposure to diverse physical activities provides opportunities to explore different ideas and practical problem-solving within motor tasks, fostering the development of self-regulation skills. Moreover, studies have shown that teaching children fundamental relational concepts improves performance on standardized achievement tests. These basic relational concepts can be challenging for many children because they lack a fixed reference point. Conversely, some indications are that engaging in physical activities may assist in developing these cognitive abilities (Piersel and McAndrews, 1982; Zhou et al., 2000; Boehm, 2013).

Our study offers valuable insights into the relationship between motor skills and cognitive abilities in preschool children, specifically constructive and conceptual thinking. We found that some of motor coordination and physical fitness tests, like shifting platforms and flexibility, predict constructive and conceptual thinking. Activities requiring high motor coordination, involving aspects like timing, proficiency, speed, and even combining timing and strength, significantly influenced both constructive and conceptual thinking, even as early as preschool age.

Although our study’s results pointed to significant links between specific motor performance and cognitive abilities, there is still more room to understand psychomotor development in preschool age. Firstly, children at this developmental stage exhibit significant variability in motor skills. Establishing clear and universal relationships between individual skills and cognitive factors is challenging. Secondly, Toomela (2016) emphasizes the importance of “semiotic mediation” in influencing how motor skills impact cognitive abilities. This suggests that other cognitive factors beyond the motor skill itself might play a role in how it affects thinking. Therefore, a comprehensive understanding of constructive and conceptual thinking requires going beyond individual motor skills and considering broader cognitive factors and the specific types of motor activities children are exposed to. This could involve investigating how motor skill practice’s duration, intensity, and context might moderate the observed relationships.

More research is necessary to fully understand the complex relationship between motor coordination, physical fitness, and cognitive development in children, particularly concerning their academic performance. By delving deeper, we can equip educators and stakeholders with valuable insights to develop engaging and creative physical activities in and outside the classroom. Specifically, focusing on innovative and challenging physical tasks during key developmental stages could foster qualities like self-confidence, memory, and anticipation skills, ultimately contributing to improved academic achievement.

### 4.1 Limitations

The present study includes tests of motor coordination (i.e., jumping laterally and shifting platforms), five tests of physical fitness (i.e., 20 m endurance run, Standing long jump, Wall toss test, shuttle run 4x5m, and Sit and reach test) which were objectively measured in children 4–5 years. We measured cognitive function focused on constructive and conceptual thinking. We used a multiple-liner regression to analyze the relationship between tests of motor coordination, physical fitness, and cognitive function precisely. Nonetheless, some limitations have to be discussed. Firstly, our study design only captures a snapshot in time, preventing us from concluding cause-and-effect or the direction of the observed associations. Additionally, the sample size was relatively small and chosen for convenience, which limits how broadly our findings can be applied.

Therefore, future research should utilize a longitudinal design. This would allow a more nuanced understanding of how these abilities interact and develop. Additionally, recruiting a more representative sample and considering factors like socio-economic background and parental attitudes toward physical activity would provide a more complete picture.

## 5 Conclusion

The results of the present study indicate that higher performance in some motor coordination and physical fitness tests is related to better constructive and conceptual thinking at preschool age. Results also suggest that physical activities demanding motor coordination and dynamic strength are potentially associated with better constructive and conceptual thinking performance, suggesting their incorporation into preschool curricula. Future research should be grounded in theory-based hypotheses, given evidence that integrated, task-relevant physical activities significantly affect learning. Subsequently, additional research is essential to determine the ideal frequency and duration of in-class physical activity programs for diverse age groups and populations (Mavilidi et al., 2015).

## Data availability statement

The original contributions presented in the study are included in the article/Supplementary materials, further inquiries can be directed to the corresponding author.

## Ethics statement

The studies involving humans were approved by Ethical Committee of the Faculty of Physical Education and Sport, Charles University. The studies were conducted in accordance with the local legislation and institutional requirements. Written informed consent for participation in this study was provided by the participants’ legal guardians/next of kin.

## Author contributions

CM: Writing – original draft, Methodology, Conceptualization. AK: Writing – original draft, Investigation, Data curation. KB: Writing – original draft, Investigation, Data curation. CC: Writing – original draft, Methodology, Formal analysis. MM: Writing – original draft, Supervision, Methodology, Conceptualization.
